# End-organ saturations correlate with aortic blood flow estimates by echocardiography in the extremely premature newborn – an observational cohort study

**DOI:** 10.1186/s12887-021-02790-1

**Published:** 2021-07-12

**Authors:** Gabriel Altit, Shazia Bhombal, Valerie Y. Chock

**Affiliations:** 1 Department of Pediatrics, Division of Neonatology, Montreal Children’s Hospital, McGill University Health Center, Montreal, Canada; 2 Division of Neonatal and Developmental Medicine, Stanford University School of Medicine and Lucile Packard Children’s Hospital Stanford, Palo Alto, USA

**Keywords:** Near infrared spectroscopy, Extreme prematurity, Regional saturation, Echocardiography, aortic blood flow

## Abstract

**Background:**

Near-infrared spectroscopy (NIRS) measures of cerebral saturation (Csat) and renal saturation (Rsat) in extreme premature newborns may be affected by systemic blood flow fluctuations. Despite increasing clinical use of NIRS to monitor tissue saturation in the premature infant, validation of NIRS measures as a correlate of blood flow is still needed. We compared echocardiography (ECHO) derived markers of ascending aorta (AscAo) and descending aorta (DesAo) blood flow with NIRS measurements obtained during the ECHO.

**Methods:**

Newborns < 29 weeks’ gestation (2013–2017) underwent routine NIRS monitoring. Csat, Rsat and systemic saturation at the time of ECHO were retrospectively analyzed and compared with Doppler markers of aortic flow. Renal and cerebral fractional tissue oxygen extraction (rFTOE and cFTOE, respectively) were calculated. Mixed effects models evaluated the association between NIRS and Doppler markers.

**Results:**

Forty-nine neonates with 75 Csat-ECHO and 62 Rsat-ECHO observations were studied. Mean post-menstrual age was 28.3 ± 3.8 weeks during the ECHO. Preductal measures including AscAo velocity time integral (VTI) and AscAo output were correlated with Csat or cFTOE, while postductal measures including DesAo VTI, DesAo peak systolic velocity, and estimated DesAo output were more closely correlated with Rsat or rFTOE.

**Conclusions:**

NIRS measures are associated with aortic blood flow measurements by ECHO in the extremely premature population. NIRS is a tool to consider when following end organ perfusion in the preterm infant.

**Supplementary Information:**

The online version contains supplementary material available at 10.1186/s12887-021-02790-1.

## Background

Extremely premature newborns undergo significant physiologic changes in their immediate post-natal life, with potential hemodynamic instability in the context of persistent fetal shunts. Neonatal care in this vulnerable population aims at maintaining adequate organ perfusion in the setting of changing physiology. Near infrared spectroscopy (NIRS) is a promising technology allowing for real-time monitoring of tissue oxygenation as a surrogate for end-organ perfusion. However, validation of NIRS measures in the premature population as a correlate of blood flow is needed, especially in the context of postnatal delays in transition, such as presence of a patent ductus arteriosus (PDA). Specifically, premature newborns are at risk of diastolic steal effect, which may adversely alter the post-ductal aortic blood flow [1].

NIRS monitoring estimates venous-weighted percentage of saturated hemoglobin in the monitored tissue [2–4]. Regional saturations are affected by organ perfusion, hemoglobin concentration, arterial oxygen content, oxygen extraction and oxygen consumption. Perfusion is a reflection of local blood flow, which is dependent on cardiac output, as well as vascular distribution, filling and tone. As such, cerebral oxygen saturation (Csat) and renal oxygen saturation (Rsat) monitored by NIRS may indicate underlying perfusion when all other factors remain stable. Csat is primarily affected by pre-ductal systemic perfusion and cerebral oxygen extraction, while the Rsat is impacted by post-ductal diastolic steal, underlying renal oxygen extraction and altered systemic output. Csat correlates with cerebral perfusion [2, 5] and decreased values have been associated with neurological injury in premature newborns [6–8]. We recently published that the velocity time integral (VTI) of the descending aortic flow by echocardiography (ECHO), a marker of stroke distance, correlated with Rsat at the time of the measurement in a population of single ventricle infants [9]. However, limited data exist on the correlation between ultrasound markers of perfusion and cerebral/renal NIRS values in the premature neonate. We sought to compare ECHO derived markers of aortic blood flow with end-organ saturation measurements obtained at the time of ECHO acquisition in extremely premature neonates. We hypothesized that in preterm infants, especially in first week of life when the duct is usually patent, resulting in diastolic runoff, Csat would reflect ECHO-derived markers of aortic pre-ductal blood flow and Rsat would reflect those of post-ductal flow.

## Methods

### Subjects

We conducted a retrospective review of all premature newborns < 29 weeks of estimated gestational age at birth with available NIRS recordings and admitted from May 2013 to August 2017 at a single institution (Lucile Packard Children’s Hospital, Stanford). Extremely premature newborns were routinely monitored with NIRS in the first 7 days of life per clinical guidelines beginning in May 2013. Csat and Rsat were recorded and stored in a secure database. Patients were excluded if ECHO was not performed during the NIRS monitoring period or if a congenital heart defect was present (except patent ductus arteriosus [PDA] or atrial or ventricular septal defects). Study data were extracted from the electronic medical record and from the neonatal NIRS database, and managed using REDCap electronic data capture tools hosted at the Stanford Center for Clinical Informatics [10]. This study was approved by the Stanford University Institutional Review Board. The study has been granted an exemption from requiring informed consent. All methods were performed in accordance with the Declaration of Helsinki.

### NIRS measures and SpO2

Csat and Rsat levels were measured using the INVOS 5100 NIRS monitor (Medtronic, Minneapolis, MN). As previously described by our group [9, 11], neonatal sensors were routinely applied shortly after birth on the central forehead and on either the right or left posterior flank above the iliac crest and below the costal margin (T10-L2) [11]. Csat and Rsat were extracted from our NIRS database, which contains prospectively collected NIRS values recorded at 30 s intervals. Systemic saturation (SpO2) values at the beginning of the ECHO were collected from nursing flowsheets. ECHO scans were stored on the picture archiving and communication system (PACS). Time at the start of the ECHO was retrieved from the images stored on the Syngo Dynamics workstation (Siemens Medical Solutions, USA). The Csat and Rsat values in the hour before and after the start of the ECHO were averaged. Cerebral and renal fractional tissue oxygen extraction (cFTOE and rFTOE, respectively), calculated as (SpO2 – [Csat or Rsat])/SpO2, was similarly collected as a measure of oxygen extraction by the brain or kidneys [3]. Clinicians were not blinded to Csat and Rsat levels, but no management guidelines based on NIRS values were in place other than notification of a medical provider if Csats were sustained < 50%.

### Echocardiographic measures

**Fig. 1:**
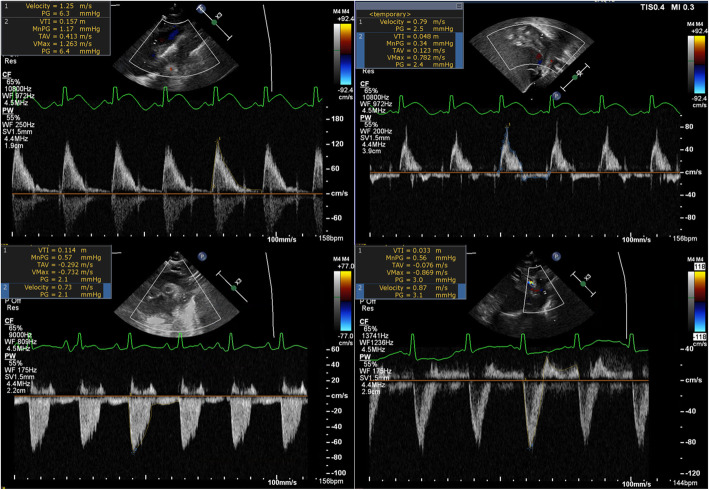
Pulse-wave Doppler envelopes in the Aorta. Legend: Peak systolic velocity and velocity time integral of the spectral envelope obtained by pulse-wave Doppler at various level in the Aorta. Panel **a**: Ascending aorta in the suprasternal view; **b**: Descending Aorta in the subcostal view (retrograde flow in diastole); **c** Post-ductal descending aorta in the suprasternal view (anterograde flow in diastole); **d** Post-ductal descending aorta in the suprasternal view (retrograde flow in diastole)

Every ECHO available during NIRS monitoring was analyzed for each patient. Methodology was similar to previous reports by our group [9, 12]. Briefly, stored images on the institutional image server were acquired according to the American Society of Echocardiography guidelines [13]. Images were acquired using Philips iE33 and Philips EPIQ 7 (Philips Medical Systems, Bothell, USA). Our standard neonatal echocardiography protocol does not involve sedation. Each ECHO parameter was prospectively measured from stored images on a Syngo Dynamics workstation (Siemens Medical Solutions, USA) by an investigator trained in pediatric ECHO (GA) and masked to NIRS saturation values. Pulsed-wave (PW) Doppler envelope at various levels in the aorta were analyzed when available (Fig. 1). Data extracted from the supra-sternal view included: ascending aorta PW-Doppler and descending (post-ductal) aorta PW-Doppler. Data extracted from the subcostal view included: descending aorta PW-Doppler. From these PW-Doppler envelopes, peak systolic velocity and velocity time integral (VTI) of the entire cardiac cycle (systolic and diastolic phase) were extracted. The diastolic VTI was considered to be zero in the absence of diastolic flow and considered to be negative when diastolic flow was retrograde. The VTI is an estimated measure of the stroke distance at the sampled area [14]. The diameter of the ascending (suprasternal view) and post-ductal descending aorta (suprasternal and subcostal views) were measured from bidimensional gray-scale images. Aortic outputs were calculated based on the respective VTI and heart rate at PW-Doppler sampling using the same formula: (Aorta diameter)/2)^2^ x π x heart rate / weight [15, 16].

### Clinical outcomes

Urine output was measured for the 8 h before the beginning of the ECHO study and expressed as mL/kg/hour (based on the daily weight). Hemoglobin, hematocrit and blood gas values (pH, pCO2, base excess) were recorded when collected within 24 h of the ECHO. Blood pressure was recorded prior to start of the ECHO.

### Statistics

Descriptive statistics were used, including mean (with standard deviations [SD]) and median (interquartile range [IQR]) for continuous variables, and counts (proportions) for categorical variables. Student t-test and Wilcoxon-Mann-Whitney test compared continuous variables for parametric and non-parametric variables, respectively. Average Csat and Rsat in the hour prior to the ECHO were compared to average Csat and Rsat in the hour after the start of the ECHO using paired Student t-test. Csat and Rsat data were correlated with ECHO parameters. Analysis accounted for the non-independent data structure (multiple ECHO measurements for some subjects at various dates) with linear mixed effect models with random intercepts/slope (for continuous variables) and generalized estimating equations (for binary categorical outcomes). The level of significance was set at 0.05 for all comparisons. Statistical analysis was done with Stata/SE 14.2 (Texas, USA).

## Results

From May 2013 to August 2017, 97 premature patients < 29 weeks gestation at birth were identified in the NIRS database with either Csat or Rsat monitoring results available. Of these, 50 infants had an available ECHO at the time of NIRS monitoring, of which 1 infant was excluded due to concomitant diagnosis of Tetralogy of Fallot. The final cohort included 49 neonates with 75 Csat-ECHO and 62 Rsat-ECHO observations available for analysis. During NIRS monitoring, a single ECHO was available in 30 patients, two ECHOs in 11 patients, three ECHOs in 7 patients and four ECHOs in 1 patient. Mean gestational age at birth was 25.8 ± 1.3 weeks, with a mean post-menstrual age of 28.3 ± 3.8 weeks at time of ECHO.

Demographic variables are shown in Table 1 and clinical variables pertaining to the ECHO-NIRS dyads in Table 2. There were no significant differences in clinical variables between those with Csat-ECHO measures and those with Rsat-ECHO measures. For those with available Rsat measurements, the average urine output was of 3.9. ± 1.7 mL/kg/min prior to ECHO. The average Rsat was of 58.6 ± 12.6% and the average rFTOE was 37.7 ± 13.3%. There was no difference between the Rsat in the hour following the onset of the ECHO compared to the Rsat in the hour preceding the ECHO (58.6 ± 12.6% vs 58.5 ± 12.3% vs, *p* = 0.90). For those with available Csat measurements, the average Csat during ECHO was 65.8 ± 8.6% and the average cFTOE was 30.1 ± 8.7%. Similarly, there was no difference between the Csat in the hour following the onset of the ECHO with the Csat in the hour preceding the ECHO (65.8 ± 8.6% vs 66.1 ± 9.1%, *p* = 0.43). The presence of an umbilical arterial line (UAL), all placed in a high-position (T6-T10), was not associated with a difference in Rsat at the start of echocardiography (54.1 ± 12.3% vs 58.6 ± 19.5%, *p* = 0.31).

**Table 1:** Demographic information

*n* = 49 newborns	
Male	19 (38%)
Inborn	38 (78%)
C-Section delivery	38 (78%)
Gestational age at birth in weeks^a, b^	25.8 (1.3)
	25.6 (23.7–28.3)
Birth weight (grams)^a, b^	805.4 (217.8)
	770 (520–1230)
APGAR at 5 minutes^b^	7 (3–9)
Antenatal steroids	Complete: 27 (56%)
	Partial: 16 (33%)
	None: 5 (10%)
Maternal Hypertension	10 (20%)
Multiple pregnancy	20 (41%)
Small for gestational age	3 (6%)

Expressed as count (proportion) or ^a^mean (standard deviation) and ^b^median (IQR). Small for gestational age defined as < 10% percentile of birth weight for gestational age as per Fenton growth chart (41)

**Table 2:** Clinical variables regarding Csat/Rsat -dyads

	Csat-ECHO dyad (*n* = 75)	Rsat-ECHO dyad (*n* = 62)
Days of life at ECHO	8 (5–15)	8 (5–15)
Post-Menstrual age at ECHO (weeks)	28.2 (3.8)	28.3 (3.8)
Systolic BP at ECHO (mmHg)	53 (11)	53 (11)
Diastolic BP at ECHO (mmHg)	27 (8)	28 (8)
SpO2 (%)	94.2 (5.0)	94.5 (5.0)
Umbilical arterial line present at ECHO	38 (51%)	34 (52%)
Hemoglobin at ECHO (g/dl)	13.0 (11.8–14.1)	12.5 (11.6–13.9)
Hematocrit at ECHO (%)	38.2 (5.4)	37.3 (4.5)
Weight at ECHO (g)	810 (670–966)	820 (680–966)
pH at ECHO	7.26 (0.07)	7.27 (0.08)
pCO2 at ECHO (mm Hg)	53.3 (10.3)	52.8 (10.4)
PDA present on ECHO	68 (91%) 60 Left to Right 8 Bidirectional	57 (92%); 50 left to right 7 Bidirectional
PDA size at ECHO (mm)	2.1 (0.1)	2.1 (0.1)
Diastolic retrograde flow in descending aorta	45 (60%)	35 (56%)

Expressed as mean (standard deviation), median (IQR) or count (proportion)

*BP* blood pressure, *Csat* cerebral saturation by near infrared spectroscopy, *ECHO* echocardiography, *PDA* patent ductus arteriosus, *Rsat* renal saturation by near infrared spectroscopy

**Fig. 2:**
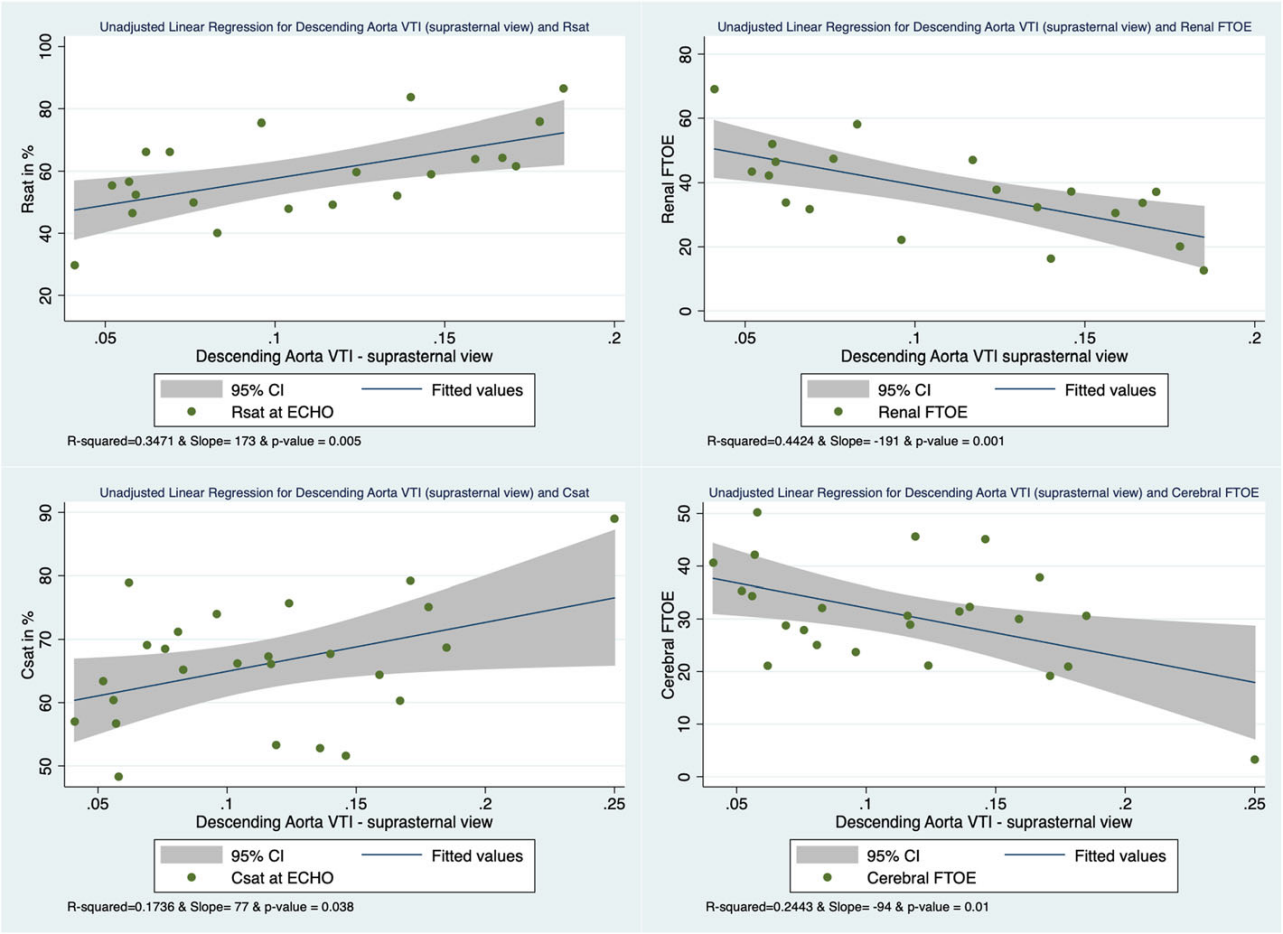
Unadjusted Linear Regression with descending aorta VTI in suprasternal view. Legend: Unadjusted linear regression indicating a correlation between Rsat and rFTOE with post-ductal descending Aortic VTI, as well as with Csat and cFTOE

Table 3 illustrates the results of the correlation between NIRS-derived measurements and ECHO-derived doppler flow measurements of the aorta. Although Rsat, Csat, rFTOE, and cFTOE did not correlate with markers of blood flow (VTI, cardiac output, and peak systolic velocity) obtained at the descending aorta and sampled with the subcostal view, the majority of these NIRS measurements did correlate with blood flow markers sampled with the suprasternal view. The unadjusted linear regressions correlating Rsat, Csat, rFTOE and cFTOE with the descending aortic VTI obtained in the suprasternal view are represented in Fig. 2. Rsat and rFTOE also were associated with the peak systolic velocity in that view. Furthermore, rFTOE, Csat and cFTOE correlated with the estimated descending aortic output measured with the suprasternal view. Csat was associated with the stroke distance estimated at the ascending aorta (suprasternal view) and estimated ascending aortic output correlated with cFTOE. Although the vast majority of the cohort had a patent ductus at evaluation, there was an association between PDA size at ECHO and Rsat/Csat values; and when stratifying for PDA presence at ECHO, we found similar results despite the decreasing number of observations (Supplemental Table A). Finally, none of the NIRS measurements were associated with the closest measurements of systolic/diastolic blood pressure, pH, hemoglobin, hematocrit, creatinine and urine output by mixed effect model.

**Table 3:** Association between NIRS measurements and ECHO-Doppler markers of Aortic flow

	Rsat at ECHO Mean 58.6% (12.6)	rFTOE at ECHO Mean 37.7% (13.3)	Csat at ECHO Mean 65.8% (8.6)	cFTOE at ECHO Mean 30.1% (8.7)
**Pre-ductal aorta – Ascending Aorta (Suprasternal View)**				
VTI Mean: 0.11 (0.03) meter	ICC: < 0.10 β(95%CI): −23(− 214–168) *p*-value: 0.81 30 observations	ICC: < 0.10 β(95%ΧΙ): 60(− 144–265) *p*-value: 0.56 30 observations	ICC: 0.75 β(95%CI): 39(−70–148) *p*-value: 0.48 36 observations	ICC: 0.48 β(95%CI): −40(− 107–114) *p*-value: 0.95 36 observations
Output Mean: 492 (136) or 500^a^ (149) mL/kg/min	ICC: < 0.10 β(95%CI): − 0.01(− 0.05–0.02) *p*-value: 0.47 30 observations	ICC: < 0.10 β(95%CI): 0.02(− 0.02–0.06) *p*-value: 0.29 30 observations	**ICC: 0.75** **β(95%CI): − 0.02(− 0.04 – − 0.002)** ***p*-value: 0.03** **36 observations**	**ICC: 0.47** **β(95%CI): 0.02(.01–0.04)** ***p*-value: 0.006** **36 observations**
Peak systolic velocity of the ascending Aorta (suprasternal view) Mean: 0.91 (0.21) or 0.93 (0.21)^a^	ICC: < 0.10 β(95%CI): −8(− 33–17) *p*-value: 0.54 30 observations	ICC: < 0.10 β(95%CI): 13(− 14–40) *p*-value: 0.33 30 observations	ICC: 0.66 β(95%CI): − 6(− 20–8) *p*-value: 0.39 36 observations	ICC: 0.48 β(95%CI): 12(− 2–26) *p*-value: 0.09 36 observations
**Post-ductal aorta - Descending Aorta (subcostal view)**				
VTI Mean: 0.04 (0.02) meter	ICC: 0.12 β(95%CI): 4(−137–144) *p*-value: 0.96 56 observations	ICC: 0.95 β(95%CI): − 27(− 177–123) *p*-value: 0.72 55 observations	ICC: 0.61 β(95%CI): 46(− 33–124) *p*-value: 0.25 69 observations	ICC: 0.50 β(95%CI): −47(− 132–38) *p*-value: 0.28 68 observations
Output Mean: 126 (56) or 122^a^ (53) mL/kg/min	ICC: 0.11 β(95%CI): 0.01(−0.05–0.07) *p*-value: 0.85 56 observations	ICC: 0.02 β(95%CI): − 0.02(− 0.08–0.05) *p*-value: 0.63 55 observations	ICC: 0.59 β(95%CI): 0.01(− 0.03–0.04) *p*-value: 0.64 69 observations	ICC: 0.57 β(95%CI): 0.01(− 0.03–0.04) *p*-value: 0.74 68 observations
Peak systolic velocity Mean: 0.52 (0.20) or 0.51^a^ (0.19) m/s	ICC: 0.11 β(95%CI): −1(− 18–15) *p*-value: 0.87 56 observations	ICC: 0.04 β(95%CI): 0.1 (− 18–19) *p*-value: 0.99 55 observations	ICC: 0.60 β(95%CI): 3(− 5–12) *p*-value: 0.44 69 observations	ICC: 0.53 β(95%CI): − 1(− 11–9) *p*-value: 0.84 68 observations
**Post-ductal aorta - Descending Aorta (suprasternal view)**				
VTI Mean 0.11 (0.05) meter	**ICC: 0.83** **β(95%CI): 180(108–252)** ***p*-value: < 0.001** **21 observations**	**ICC: 0.71** **β(95%CI): − 188(− 264 – − 111)** ***p*-value: < 0.001** **20 observations**	**ICC: 0.37** **β(95%CI): 71(7–135)** ***p*-value: 0.03** **25 observations**	**ICC < 0.10** **β(95%CI): − 94(− 161 – − 28)** ***p*-value: 0.005** **24 observations**
Output Mean: 279 (122) or 273^a^ (124) mL/kg/min	ICC: 0.23 β(95%CI): 0.03(−0.01–0.08) *p*-value: 0.16 21 observations	**ICC: 0.03** **β(95%CI): − 0.05(− 0.10 – − 0.01)** ***p*-value: 0.03** **20 observations**	**ICC: 0.96** **β(95%CI): 0.018 (0.002–0.034)** ***p*-value: 0.02** **25 observations**	**ICC: 0.98** **β(95%CI): − 0.03(− 0.04 – − 0.01)** ***p*-value: < 0.001** **24 observations**
Peak systolic velocity Mean: 0.94 (0.32) or 0.97^a^ (0.33) m/s	**ICC: 0.47** **β(95%CI): 23(7–38)** ***p*-value: 0.004** **21 observations**	**ICC: 0.38** **β(95%CI): − 37(− 50 – − 23)** ***p*-value: < 0.001** **20 observations**	ICC: 0.92 β(95%CI): 7(−2–16) *p*-value: 0.129 25 observations	ICC: 0.88 β(95%CI): − 8(− 19–3) *p*-value: 0.17 24 observations

Linear mixed effect models with random intercepts (for continuous variables). Mean expressed with (standard deviation). Statistically significant correlations are in bold type. Outputs in mL/kg/min. Peak velocities in meter/second

*Ao* aorta, *β* β coefficient, *Csat* cerebral saturation, *ECHO* echocardiography, *FTOE* fractional tissue oxygen extraction, *ICC* intraclass correlation, *Rsat* renal saturation, *VTI* velocity time integral in meters

^a^ECHO parameters mean (SD) for corresponding renal NIRS measures or cerebral NIRS measures

## Discussion

In this retrospective study of NIRS measures in extremely premature neonates, we found an association between end organ saturations measured during ECHO with estimates of aortic flow by Doppler. As physiologically expected, markers of pre-ductal aortic flow correlated with Csat and those of post-ductal aortic flow with Rsat.

We previously described that descending abdominal aortic flow by subcostal view correlated with Rsat measures in a congenital heart disease term population [9]. In this current extreme premature population, markers of aortic blood flow in the suprasternal view were correlated with NIRS measures of end-organ saturations. Csat correlated with both ascending aortic measurements (pre-ductal) and descending aortic measurements while Rsat correlated with descending aortic measurements only. Other authors have described a correlation between cerebral saturation and estimated cardiac output in the extreme premature neonatal population [17]. In our study, measurements in the subcostal view were not associated with NIRS measurements. This may be explained by the increased angle of insonation in premature newborns, when evaluating the descending aorta in the subcostal window, compared to a term newborn where one may have more amplitude of manipulation of the probe. During Doppler acquisition, angle of insonation between the sampled vessel and the ultrasound beam must be minimized to avoid imprecision in estimating the Doppler shift [18, 19]. As such, the beam should interrogate the corresponding flow in a parallel fashion, as much as feasible [20]. Suprasternal view minimizes the angle of insonation for Doppler acquisition in the descending aorta, since it is aligned with the flow. Indeed, a previous report indicated that flow velocities for the descending aorta are best sampled from the suprasternal window [21]. Finally, estimation of stroke distance by VTI has been advocated as a simple and reproducible measurement of blood flow [22], which can be used to monitor the hemodynamic changes in critically ill patients [23] and, in select populations, has been associated with patient outcomes [24, 25]. It has previously been described as a marker of lower body perfusion in the pediatric and neonatal population [26]. As such, our data suggest that a suprasternal approach be considered when interrogating the post-ductal aorta to assess VTI in the premature neonate.

NIRS is a promising tool to monitor changes in aortic blood flow, as may be seen with a significant PDA in the extremely premature neonate. Other clinical markers of perfusion commonly used in the clinical setting, including blood pressure, may not detect subtle changes [27, 28] and have not been correlated with echocardiographic markers of blood flow in preterm infants [28]. While indicators of aortic blood flow were associated with Rsat and Csat measurements in our study, they were not associated with other routinely used markers of end-organ hypoperfusion in clinical practice: blood pressure, urine output, pH and hemoglobin. While these traditional markers may be disturbed when tissue ischemia is ongoing [29, 30], they may be imperfect as early detectors of hypoperfusion. Low Csat values have been associated with increased likelihood for severe intraventricular hemorrhage or periventricular leukomalacia in premature newborns [6–8]. Altered blood flow patterns may be associated with abnormal Csat values and contribute to the hemodynamic changes involved in the pathophysiology of neurological injury. Rsat values are usually higher than Csat values in term infants. However, in our population, Rsat values were lower, possibly reflecting the high incidence of a left-to-right PDA on the ECHO scans that were included. In the Rsat-ECHO dyads, 92% had a PDA with a significant proportion showing a diastolic steal (56% retrograde flow in the descending aorta), possibly compromising the renal perfusion and explaining the lower Rsat. Our group has previously described an association between a hemodynamically significant PDA and a tendency for a low Rsat [11]. In our cohort, we also found an association between PDA size and Rsat values (Supplemental Table A). Our data suggest that Csat and Rsat, as measured with NIRS, can be utilized as a correlate of pre- and post-ductal systemic blood flow. This adds to the small body of literature associating NIRS measures to markers of perfusion in the pediatric setting [9, 31–33].

Several methodologic issues regarding the stability of NIRS measures during concurrent ECHO image acquisition and infusion through an indwelling UAL were investigated. The regional oxygen saturation/extraction values were similar in the hour prior and after the ECHO, suggesting that the image acquisition did not affect oxygen utilization. Furthermore, in our cohort, we did not detect any association between NIRS values and the presence of an UAL. However, the granularity of the NIRS measurements may not have detected subtle blood flow differences secondary to the presence of the line, and time periods of blood sampling from the UAL were not specifically captured. The stability of NIRS measures as described in this study may help to inform future design of simultaneous NIRS and ECHO studies in the preterm infant.

Our study was retrospective, single center and descriptive with a significant risk of being underpowered to detect meaningful associations. Additional limitations include lack of NIRS data for some infants with prematurity and the fact that clinicians were not blinded to the NIRS values. Also, not all infants had an ECHO during NIRS monitoring, and as such, were excluded from the analysis. Hence, the current cohort most likely represents the sickest premature newborns from a clinical perspective, potentially introducing selection bias that would limit generalizability. Arterial oxygen tension (PaO2) at the time of NIRS monitoring could have provided additional information and added accuracy compared to the use of the systemic oxygen saturation but were not consistently available in all patients. Creatinine measurements were only available for some infants and timing of collection was variable since retrospectively extracted from the chart. Precise position of renal parenchyma by ultrasound was not confirmed prior to sensor placement. However, previous studies have evaluated by ultrasound the position of the kidney in newborns and described its location as between the costal margin and the iliac crest [34–36]. ECHO was limited by the use of multiple scanning platforms, multiple professionals acquiring the images, absence of some ECHO markers, or limited views in several patients. Indeed, as outlined in Table 3, some ECHO markers were missing in the included ECHOs (potentially introducing a measurement bias) and many newborns had only some of the ECHO markers available for analysis (number of observations available were presented). ECHO measures were not analyzed for intra-reader or inter-reader variability, although our methodology for VTI reproducibility has been validated in a previous publication by our group [24], and by other authors [22, 37, 38]. Furthermore, output estimation by ECHO has limitations regarding the assumption that the cross-sectional area has a circular shape and is calculated solely from the linear diameter measurement of the corresponding structure. However, a MRI study has indicated a strong correlation with ECHO-derived measurements of output, based on this methodology [39]. Finally, the ECHOs were not performed at standardized time-points.

## Conclusions

Regional NIRS values are associated with aortic blood flow measurements by ECHO in the extremely premature population. Future studies should investigate if therapeutic approaches to maintain adequate end-organ saturations using NIRS monitoring may also favorably impact blood flow distribution and improve outcomes in the extremely premature population.

## Supplementary Information

**Additional file 1: Supplemental Table A.** Associations in those with and without PDA at ECHO.

## Data Availability

The derived data generated in this research will be shared on reasonable request to the corresponding author.

## Abbreviations

Csat: Cerebral regional oxygen saturation level
ECHO: Echocardiography
FTOE: Fractional tissue oxygen extraction
NIRS: Near-infrared spectroscopy
PDA: Patent ductus arteriosus
PVR: Pulmonary vascular resistance
Rsat: Renal regional oxygen saturation level
SpO2: Systemic oxygen saturation
UAL: Umbilical arterial line
VTI: Velocity time integral
