# Supplementary material for: End-organ saturations correlate with aortic blood flow estimates by echocardiography in the extremely premature newborn – an observational cohort study
Source: BMC Pediatr. 2021 Jul 12;21:312. doi: 10.1186/s12887-021-02790-1 (PMC8274006; doi:10.1186/s12887-021-02790-1)
Supplement: Supplementary file 1 — Additional file 1: Supplemental Table A. Associations in those with and without PDA at ECHO. [file 12887_2021_2790_MOESM1_ESM.docx]

**Supplemental Table A – Associations in those with and without PDA at ECHO**

|  | | **Rsat at ECHO**  **Mean 58.6% (12.6)** | **rFTOE at ECHO**  **Mean 37.7% (13.3)** | **Csat at ECHO**  **Mean 65.8% (8.6)** | **cFTOE at ECHO**  **Mean 30.1% (8.7)** |
| --- | --- | --- | --- | --- | --- |
| **Measurements in those without PDA** | | | | | |
| **Pre-ductal aorta – Ascending Aorta (Suprasternal View)** | | | | | |
| Output  mL/kg/min | | Not significant  3 observations | **β(95%CI): 0.15(0.14 –0.16)**  **p-value: <0.001**  **3 observations** | **β(95%CI): 0.11(0.05 – 0.17)**  **p-value: <0.001**  **3 observations** | Not significant  3 observations |
| **Post-ductal aorta - Descending Aorta (suprasternal view) in those with PDA** | | | | | |
| VTI  In meter | | **β(95%CI): 445(371 – 520)**  **p-value: <0.001**  **4 observations** | **β(95%CI): -503(-727 – -280)**  **p-value: <0.001**  **4 observations** | Not significant  5 observations | **β(95%CI): 124(16 – 232)**  **p-value: 0.03**  **5 observations** |
| Output  in mL/kg/min | | **β(95%CI): 0.12(0.02 – 0.21)**  **p-value: 0.02**  **4 observations** | **β(95%CI): -0.16(-0.25 – -0.06)**  **p-value: 0.001**  **4 observations** | Not significant  5 observations | Not significant  5 observations |
| Peak systolic velocity  in m/s | | **β(95%CI): 17(11 – 24)**  **p-value: <0.001**  **4 observations** | **β(95%CI): -21(-29 – -15)**  **p-value: <0.001**  **4 observations** | Not significant  5 observations | Not significant  5 observations |
| **Measurements in those with PDA** | | | | | |
| PDA size in cm  Mean: 0.21 (0.09) or 0.21 (0.08)** | **Mean: 0.21 (0.09)**  **ICC: 0.41**  **β coefficient: -39.56**  **p-value: 0.03**  **95%CI: -74.10 – -5.02**  **56 observations** | **Mean: 0.21 (0.09)**  **ICC: 0.31**  **β coefficient: 45.75**  **p-value: 0.02**  **95%CI: 8.97 – 82.52**  **55 observations** | **Mean: 0.21 (0.08)**  **ICC: 0.53**  **β coefficient: -25.87**  **p-value: 0.046**  **95%CI: -51.24 – -0.51**  **67 observations** | Mean: 0.21 (0.08)  ICC: 0.51  β coefficient: 26.31  p-value: 0.05  95%CI: -0.01 – 52.62  66 observations |  |
| **Pre-ductal aorta – Ascending Aorta (Suprasternal View)** | | | | | |
| Output  mL/kg/min | | Not significant  27 observations | Not significant  27 observations | **β(95%CI): -0.02(-0.04 – 0.004)**  **p-value: 0.01**  **33 observations** | **β(95%CI): 0.03(0.01 – 0.04)**  **p-value: 0.004**  **33 observations** |
| **Post-ductal aorta - Descending Aorta (suprasternal view) in those without PDA** | | | | | |
| VTI  In meter | | **β(95%CI): 187(114 – 260)**  **p-value: <0.001**  **17 observations** | **β(95%CI): -192(-272 – -112)**  **p-value: <0.001**  **16 observations** | **β(95%CI): 93 (24 – 162)**  **p-value: 0.008**  **20 observations** | **β(95%CI): -107 (-181 – -34)**  **p-value: 0.004**  **19 observations** |
| Output  in mL/kg/min | | Not significant  17 observations | Not significant  16 observations | **β(95%CI): 0.02(0.01 – 0.04)**  **p-value: 0.009**  **20 observations** | **β(95%CI): -0.03(-0.04 – -0.01)**  **p-value: <0.001**  **19 observations** |
| Peak systolic velocity  in m/s | | **β(95%CI): 25(3 – 46)**  **p-value: 0.02**  **17 observations** | **β(95%CI): -54(-73 – -35)**  **p-value: <0.001**  **16 observations** | **β(95%CI): 10(0.7 – 20)**  **p-value: 0.03**  **20 observations** | Not significant  19 observations |
| Linear mixed effect models with random intercepts (for continuous variables). Mean expressed with (standard deviation). Statistically significant correlations are in bold type. Outputs in mL/kg/min. Peak velocities in meter/second. β (β coefficient), Csat (cerebral saturation), ECHO (echocardiography), FTOE (fractional tissue oxygen extraction), ICC (intraclass correlation), Rsat (renal saturation), VTI (velocity time integral in meters). **ECHO parameter mean (SD) for corresponding renal NIRS measures or cerebral NIRS measures | | | | | |
